# Prebiotic Strategies to Manage Lactose Intolerance Symptoms

**DOI:** 10.3390/nu16071002

**Published:** 2024-03-29

**Authors:** Gloria Angima, Yunyao Qu, Si Hong Park, David C. Dallas

**Affiliations:** 1 Department of Food Science & Technology, Oregon State University, Corvallis, OR 97331, USA; angimag@oregonstate.edu (G.A.); yunyao.qu@oregonstate.edu (Y.Q.); 2 Nutrition Program, School of Nutrition and Public Health, College of Health, Oregon State University, Corvallis, OR 97331, USA

**Keywords:** *Bifidobacterium*, hypolactasia, lactase-phlorizin hydrolase, beta-galactosidase, galactooligosaccharides

## Abstract

Lactose intolerance, which affects about 65–75% of the world’s population, is caused by a genetic post-weaning deficiency of lactase, the enzyme required to digest the milk sugar lactose, called lactase non-persistence. Symptoms of lactose intolerance include abdominal pain, bloating and diarrhea. Genetic variations, namely lactase persistence, allow some individuals to metabolize lactose effectively post-weaning, a trait thought to be an evolutionary adaptation to dairy consumption. Although lactase non-persistence cannot be altered by diet, prebiotic strategies, including the consumption of galactooligosaccharides (GOSs) and possibly low levels of lactose itself, may shift the microbiome and mitigate symptoms of lactose consumption. This review discusses the etiology of lactose intolerance and the efficacy of prebiotic approaches like GOSs and low-dose lactose in symptom management.

## 1. Introduction

Milk and dairy products are widely consumed and contribute an array of essential macro- and micronutrients to the human diet [1]. Milk production from mammary glands is a unique characteristic of mammals that evolved over more than 300 million years [2]. Mammalian infants exclusively rely on milk for nourishment in the early postnatal period [2]. Lactose, a disaccharide with the chemical composition galactose-β-1,4-glucose, is the main carbohydrate in milk and is a key energy source for infants [3,4].

Lactose digestion occurs in the small intestine. The lactose β-1–4 bond is enzymatically cleaved, releasing the monosaccharides glucose and galactose, which can be absorbed across the intestinal epithelium via transport proteins [5]. This digestion is induced by a β-galactosidase enzyme called lactase-phlorizin hydrolase, commonly known as lactase [6]. Lactase production by small intestinal enterocytes in the brush border begins in mammals during gestation and peaks at birth [7,8]. In contrast to all other wild mammals who stop expressing lactase post-weaning, a considerable proportion (approximately 25–35%) of the human population maintains lactase activity into adulthood, called lactase persistence (LP) [6,9]. The evolution of lactase persistence in humans aligns with the rise of dairying historically and is thought to be the result of positive selection for being able to gain the nutritional benefits of milk post-weaning [10]. Approximately 65–75% of humans are lactase non-persistent (LNP), in that they do not express adequate lactase post-weaning, resulting in gastrointestinal symptoms, such as bloating, flatulence, diarrhea and nausea, when lactose is consumed [3,11]. Lactose intolerance (LI) is defined as the display of such gastrointestinal symptoms due to inefficient lactose digestion, also called lactose malabsorption.

Dietary lactose does not influence the production of intestinal lactase. Studies confirm that lactase levels in LNP individuals remain constant regardless of dietary lactose consumption or avoidance [11]. Thus, while colonic bacteria can adapt to improve lactose digestion, the innate lactase enzyme expression is not affected by lactose intake.

The main management strategies for LI are reducing or eliminating the consumption of lactose-containing foods and consuming supplemental lactase enzymes with lactose-containing meals. Lactose consumption in people with LNP does not increase lactase production [11]. Consumption of prebiotics or lactose may, however, lead to adaptations in the microbiome that may contribute to LI symptom management in people with LNP. A limited number of studies suggest that prebiotics, including galactooligosaccharides (GOS) and potentially low doses of lactose itself, can enhance the proliferation of lactose-metabolizing microorganisms, such as *Bifidobacterium* and *Lactobacillus* [12,13,14,15,16,17], and decrease LI symptoms [17]. This review focuses on primary lactose intolerance caused by the cessation of lactase production post-weaning, which is the principal cause of lactose malabsorption. The review examines the mechanisms of LI, its genetic basis, diagnostic approaches, and the emerging use and mechanisms of prebiotics in managing LI symptoms.

## 2. Lactose Biosynthesis and Lactase

Lactose, predominantly found in milk, is relatively scarce in other human-consumed foods, with few exceptions, such as certain plant species like forsythia flowers [18]. The biosynthesis of lactose is energetically intensive. The enzyme galactosyl transferase catalyzes the transfer of a uridine triphosphate (UDP)-bound galactose to an acceptor glucose [18,19]. Hormonal shifts, including an increase in prolactin and a decrease in progesterone, are essential for inducing lactose synthesis [18].

In the small intestine of infants, lactase cleaves the lactose β-1–4 bond, releasing glucose and galactose, which are then absorbed and metabolized [5]. The energy-intensive mechanisms of lactose synthesis and lactase-dependent digestion imply a selective advantage in mammalian evolution [20]. Lactase regulation has been hypothesized to play a role in promoting weaning and optimizing birth spacing by initiating the reduction inlactase expression over time, which leads to increases in LI symptoms, thus helping to encourage the introduction of weaning foods to the infant [18]. The subsequent reduction in suckling frequency promotes involution (cessation of milk production) [18], which helps reverse lactation’s suppression of fertility to allow the lactating parent to conceive [18,21].

## 3. Mechanisms of Lactose Malabsorption and Intolerance

Lactose malabsorption is the inefficient digestion of lactose due to a deficiency in lactase production, known as hypolactasia. The most common type of lactose malabsorption is primary deficiency, which results from the loss of production of lactase post-weaning [22]. This condition is differentiated from secondary deficiency, which is induced by external factors such as gastrointestinal disorders, malnutrition or certain surgical procedures that temporarily reduce lactase levels [23]. A rarer form is the congenital lactase deficiency caused by the inheritance of two defective lactase genes [24].

When lactose goes undigested in the small intestine, it proceeds to the large intestine, which can lead to several symptoms. First, the increased concentration of lactose in the colon leads to higher osmotic pressure, and more water is drawn into the colon, which can result in diarrhea [25]. A wide array of colonic microbes can produce β-galactosidase to release glucose and galactose from lactose and then further ferment these monosaccharides [26]. Fermentation of lactose by colonic microbiota can produce gases like hydrogen, carbon dioxide and methane, leading to abdominal pain, bloating, and flatulence [3]. A variety of commensal intestinal bacteria can produce hydrogen from lactose fermentation, including various species of *Bacteroides* (e.g., *B. fragilis*, *B. thetaiotamicron*, and *B. ovatus*) and *Clostridium* (e.g., *C. perfringens*, *C. fallax*, *C. paraputrificum*, *C. histolyticum*, and *C. septicum*) [27]. Some of these species (e.g., *B. thetaiotamicron*) are also known to be able to produce β-galactosidase to degrade lactose [28]. Some bacteria, like *Veillonellaceae* can ferment a product of lactose fermentation (lactate) and produce carbon dioxide [29,30]. Methane production is predominantly carried out by methanogenic archaea, like *Methanobrevibacter smithii* [31], which are commonly present in the human gut [32], that produce methane using hydrogen and carbon dioxide from bacterial fermentation [33].

## 4. Lactase Non-Persistence and Persistence

Lactase activity is evident on the fetal intestine’s mucosal surface around eight weeks of pregnancy, increases throughout gestation and peaks at birth [34]. Post-weaning, a significant decrease or even cessation of lactase production is common in most mammals, including most humans [6]. About 65–75% of humans globally display LNP and are, therefore, intolerant to foods with large amounts of lactose [4,10]. Lactase non-persistence (LNP) occurs because of an autosomal recessive trait [35].

The onset age for reduced lactase activity varies across populations [7]. Cook et al. found that lactase activity (measured via lactose tolerance test) decreased in Ugandan children as early as six months old, and most subjects were deficient by between ages three and four [36]. In a study of 169 Chinese children 2–16 years old, lactose challenges via hydrogen breath test (HBT) were positive in 41% of subjects at age three to four and increased with age to 94% by age nine [37]. Furthermore, research on 852 Chinese children indicated lactase activity reduction (based on HBT) starting after age 2, with a peak percentage of participants with lactose malabsorption from ages 3 to 5, which then stabilized [10].

These studies demonstrate that for most people with LI, the phenotype is apparent by age five but can occur later. A subset of the human population (about 25–35%) [4,10] retains lactase activity into adulthood, a genetic adaptation known as lactase persistence (LP), which is an autosomal dominant genetic trait [38]. The correlation between LP and the domestication of milk-producing livestock, as well as milk consumption, is well-established [10,11]. Researchers hypothesize that the nutritional advantages conferred to people by the continued ability to consume dairy products post-weaning resulted in positive selection for the LP trait [39,40,41].

The distribution of these lactase phenotypes varies globally and has been described previously [10,42]. Briefly, LP is most common in northwestern Europe, and the percentage of people with LP decreases across southern and eastern Europe [43]. In India, the frequency of LP is highest in the northwest and decreases eastward [10]. The frequency of LP in Asia is typically very low (15% in China and 0% in South Korea, Vietnam, and Cambodia) [42]. LP prevalence in South America and Africa is about 50% [44]. However, LP is common in the milk-dependent pastoralist communities of the Middle East and Africa (86% in Bedouin Saudi, 88% in Ben-Amir, and 70% in the Fulani) [42,45,46]. Some pastoralist communities that use milk products have a low prevalence of LP, for example, the Dinka and the Nuer in Sudan [10]. This finding may be due to their more recent initiation of milk consumption and, thus, limited time for genetic changes that cause LP to accrue in the population [47], or because milk is mostly consumed in its fermented forms (e.g., yogurt, cheese), which have reduced lactose [5].

The LCT gene, responsible for lactase production, is located on chromosome 2q21 and is subject to regulation by cis-acting elements [34,48]. The single nucleotide polymorphism (SNP) C-13910 > T, found 13.9 kb upstream of the LCT gene transcription start site, has been associated with LP in European adults and children based on intestinal biopsy lactase activity and lactose intolerance testing [10,45,49]. This nucleotide change has been shown to affect lactase promoter activity in in vitro studies [49,50,51]. Though the –13910*T allele is likely the main causal variant for LP in Europeans, this SNP is absent from most African pastoralist communities with a high frequency of LP [38]. In African populations, LP is mediated by SNPs such as G-14009 > C, G-14010 > C, T-13915 > G, and C-13907 > G, all of which have been shown to affect lactase promoter expression in vitro [10,38,52]. Additionally, -13915*G is linked to LP in Saudi Arabia [34]. G-22018 > A is a common variant for LP in several Asian populations [53,54]. There are many other SNPs associated with LNP [42]. The presence of distinct SNPs leading to LP indicates that the trait evolved independently in different world regions [10], reflecting convergent evolution likely due to selective advantages from consuming lactose-containing dairy products post-weaning [20].

## 5. Diagnosis

The diagnosis of LI predominantly relies on self-identification of symptoms after the consumption of lactose-containing foods in individuals with LNP [55]. Clinically, five primary diagnostic methods are employed (Table 1).

The lactose tolerance test gauges lactose metabolism by measuring blood glucose concentration, with levels assessed at half-hourly intervals for three hours post-ingestion of 1–1.5 g of lactose per kg of body weight [5]. A rise exceeding 20 mg glucose/dL is indicative of lactose tolerance [25]. The lactose tolerance test has 76–96% specificity and 76–94% sensitivity [56]. This test can yield false positives in cases of rapid gastrointestinal transit or impaired glucose tolerance [3].

Genetic testing for primary LI can be conducted using techniques such as polymerase chain reaction (PCR) restriction, fragment length polymorphism, real-time PCR assays, and sequencing analysis. All known SNPs associated with LNP can be examined as an indicator [34].

Measuring lactase activity in endoscopic duodenal biopsies is another diagnostic tool for lactose intolerance (LI) [3,25]. Lactase activity in these samples is semi-quantitatively analyzed using a colorimetric reaction [57]. The Quick Lactase Test (Biohit PLC, Helsinki, Finland), as employed by Kuokkanen et al., facilitates the diagnosis of severe hypolactasia with 95% sensitivity and 100% specificity [58]. Though false positives and negatives are uncommon with the biopsy test, it is rarely used because it is expensive and invasive [24].

The hydrogen breath test (HBT) is a cost-effective, non-invasive, and simple diagnostic method for LI [57]. In people with LNP, ingested lactose is not absorbed and is fermented by colonic bacteria, leading to hydrogen gas production [59]. (However, as we will discuss, this production may vary based on microbiome composition.) This hydrogen diffuses into the blood and is exhaled via the lungs. The HBT involves measuring breath hydrogen at 30 min intervals for approximately three hours after the administration of a 25 g lactose challenge dose (equivalent to roughly 500 mL of milk) [59]. A positive result is indicated by a hydrogen increase of at least 20 parts per million over the baseline after three hours [60]. The test is typically used alongside the identification of clinical symptoms to diagnose LI [61]. To reduce the likelihood of false negatives or positives, subjects must avoid certain foods, medications and activities that can alter hydrogen levels prior to testing (e.g., fermentable carbohydrates [59], antibiotics [62], motility drugs and laxatives [63], smoking [60], and excessive exercise [63,64]). The HBT offers approximately 80% sensitivity and 70% specificity [65]; however, its ability to determine the severity of hypolactasia is limited [57].

The Gaxilose test is an innovative, non-invasive diagnostic tool to evaluate lactase activity [66]. This test involves administering gaxilose (galactose β1–4 xylose)=—a synthetic disaccharide akin to lactose, which is similarly processed by intestinal lactase into galactose and xylose. The xylose is then absorbed, and its levels can be measured in blood and urine to determine lactase activity. The specificity of this test appears to be lower than the Lactose tolerance test [53].

Employing multiple diagnostic tests for LI can enhance specificity and sensitivity. Moreover, assessing additional gases like methane, which is produced during lactose fermentation by certain gut bacteria, can further refine diagnostic accuracy [67,68]. This approach is particularly valuable as it may detect LI in the subset of the population—20% to 30%—that predominantly produces methane rather than hydrogen during bacterial fermentation [67].

## 6. Management of Symptoms

The primary management strategy for LI involves avoiding lactose-containing foods. Individuals with LI often tolerate up to 12 g of lactose in a dose, equivalent to the amount in 250 mL of milk, without symptoms; hence, lower doses are recommended for consumption [4]. Lactase supplements are available and can be consumed with dairy products to supplement digestion and reduce symptoms. Additionally, lactose-free dairy products provide an alternative to conventional dairy for those with LI [69,70,71,72].

### 6.1. The Use of Prebiotics in the Management of LI Symptoms

Currently, there is no known method to increase lactase expression in individuals with LNP [24]. A review by Forsgård (2019) indicates that 14 studies demonstrate that consuming lactose does not increase intestinal lactase production in LNP humans [11]. Some studies suggest that symptoms may be mitigated via regular consumption of prebiotics or low-level lactose [73,74].

### 6.2. Gut Microbiota and Lactose Intolerance

The gut microbiome, established postnatally, becomes relatively stable by the third year of life [75]. About 60–70% of the microbiome composition remains constant throughout life, while 30–40% can be influenced by diet, physical activity, antibiotic use, and surgical interventions [75]. A large array of gut microbial species can produce β-galactosidase to release glucose and galactose from lactose and then further ferment these monosaccharides [26]. Many of these bacteria produce gases like hydrogen, carbon dioxide, and methane from lactose fermentation, which can lead to symptoms of lactose intolerance, like abdominal pain, bloating, and flatulence [3]. Strains of *Lactobacillus* and *Bifidobacterium* also produce β-galactosidase, and metabolize lactose very efficiently [6,76]. Unlike other bacteria, *Lactobacillus* and *Bifidobacterium* do not produce gas during lactose fermentation, which could help decrease the likelihood of bloating and flatulence [27]. Moreover, the rapid fermentation capacity of *Lactobacillus* and *Bifidobacterium* may play a role in lessening osmotic diarrhea associated with LI [76]. Enhancing the colonization of the intestine with these *Lactobacillus* and *Bifidobacterium* through the intake of prebiotics could be a promising strategy to mitigate LI symptoms [13].

#### 6.2.1. Prebiotics and Galactooligosaccharides (GOS)

Prebiotics are substrates that can be selectively used by certain beneficial microorganisms and lead to their increased growth, leading to positive changes in both the composition and functionality of the microbiome [77,78]. An example prebiotic is GOS, which are oligosaccharides that are made up of galactose units linked by glycosidic bonds with a terminal glucose [73,79]. As humans lack the enzymes to break down the glycosidic bonds in GOS, they remain intact to the colon where they can be fermented by bacteria [80].

GOS is currently added to infant formulas to emulate the effects of human milk oligosaccharides (HMOs) [81] as both can be fermented by and enhance the growth of *Bifidobacteria* [82,83,84,85,86,87]. *Bifidobacteria* are associated with healthy gut function and immune system development [85,86,88,89]. Though studies typically indicate that GOS consumption increases *Bifidobacteria*, some studies indicate that a portion of the population does not have this response [77,87], and, in at least one study, non-responders had lower initial levels of *Bifidobacteria* than other participants [77].

In addition to *Bifidobacteria*, GOS supplementation can enhance the abundance of *Lactobacillus* in the gut. A study supplementing milk replacer with GOS in poorly performing piglets showed increased presence of *Lactobacillus* and *Bifidobacterium* as well as improvements in gut architecture [90]. This evidence suggests that GOS may encourage a broader spectrum of beneficial gut bacteria, including *Lactobacillus*, known for its positive role in gut health and immunomodulation. The study highlights that GOS-supplemented diets may offer comprehensive support to the gut microbiome, which could be particularly beneficial in improving gastrointestinal performance [90].

#### 6.2.2. Impact of GOS on Lactose Intolerance

Three placebo-controlled studies have explored the impact of GOS alone on the gut microbiota in individuals with LI and on the alleviation of associated symptoms [91] (Table 2). GOS intake has been found to promote the proliferation of lactose-fermenting bacteria such as *Bifidobacteria*, *Lactobacillus*, and *Faecalibacterium*, and this increase in beneficial bacteria is linked to a decrease in LI symptoms [12,13,17].

For example, a randomized, double-blind, placebo-controlled trial by Savaiano et al., demonstrated that feeding subjects with LI confirmed by HBT (*n* = 85 (GOS: 57; Placebo: 28)), GOS at 1.5 g/day, and escalating to 15 g/day over 35 days (with no dairy consumption) indicated a trend towards improvement in symptoms such as abdominal pain, cramping, bloating, and flatulence compared with the placebo group (*p* = 0.06) after lactose challenge [17]. Moreover, the increase in flatulence after a lactose challenge was significantly lower in GOS group than the placebo group (*p* = 0.04). These findings indicate the potential of GOS consumption to alleviate LI symptoms.

Samples from the Savaiano et al. study were later investigated for effects on the microbiome [12]. GOS supplementation led to an increase in lactose-metabolizing bacteria *Bifidobacteria*, *Faecalibacterium*, and *Lactobacillus* compared with baseline and with the placebo group [12]. After returning to dairy consumption for one month, there was a discernible reduction in microbial diversity and richness compared to day 0, likely due to the expanded abundance of *Bifidobacteria* and *Lactobacillus* at the expense of other genera, such as Enterobacteriaceae and *Streptococcus*, which diminished following GOS consumption. This finding aligns with other research suggesting that GOS promotes *Bifidobacteria* proliferation and a reduction in *Bacteroidetes* [85]. Moreover, there was a negative correlation observed between the abundance of Bifidobacterium and the experience of cramping and pain after dairy reintroduction to the participants’ diets.

Another randomized trial feeding low-dose GOS (5 g twice daily for 10 days, 7.5 g twice daily for 20 days), high-dose GOS (7.5 g twice daily for 10 days, 10 g twice daily for 20 days), or placebo to LI subjects (n = 377) for 30 days found a significant rise in the fecal relative abundance of various *Bifidobacterium* species in both GOS groups [13]. Moreover, after a lactose challenge on day 31, participants in the pooled GOS groups had significant reductions in cramping (*p* = 0.026) and bloating (*p* = 0.028), and a trend towards reduced abdominal pain and gas movement. After encouragement to voluntarily resume consumption of dairy products from day 32 to 60, those in the pooled GOS groups reported a significant increase in milk consumption (*p* = 0.008) compared with prior to the treatment, indicating a potential improvement in lactose tolerance.

The recent studies on GOS consumption suggest that it can increase beneficial lactose-fermenting bacteria in the gut of individuals with lactose intolerance, resulting in decreased symptom severity. The data suggest that GOS consumption might not only offer temporary relief but could also initiate a more enduring adaptation within the gut ecosystem.

#### 6.2.3. Potential Mechanism for Lactose Utilization and Symptom Reduction

**Figure 1:**
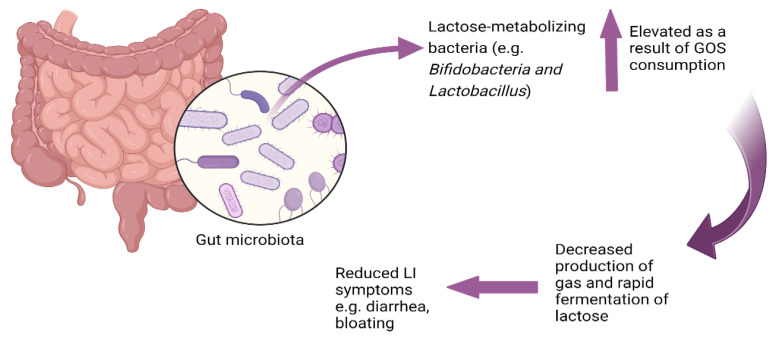
Lactose as a prebiotic: microbial adaptation to lactose consumption.

The mitigation of lactose intolerance symptoms following continuous consumption of GOS appears to be a consequence of GOS’s ability to enhance the growth of lactose-metabolizing *Bifidobacteria* and *Lactobacillus* which can ferment lactose more rapidly than other bacteria and ferment lactose without gas production (unlike many bacteria [27]), thus lowering potential symptoms of lactose intolerance. *Bifidobacteria* ferment disaccharides and oligosaccharides more readily than monosaccharides [92]. *Bifidobacteria* employ a unique carbohydrate metabolic pathway known as the bifid shunt [93], which leads to the production of lactate, acetate, and ethanol without generating gas [94]. Lactose metabolism by *Bifidobacteria* does not result in the production of gases such as hydrogen [15]. For example, metabolism of 13C-labelled lactose by *Bifidobacterium animalis* subsp. *lactis* BB-12 resulted in lactate and acetate as the primary fermentation end-products and did not result in gas production [92]. This rapid fermentation potentially reduces the osmotic pressure in the gut, thereby decreasing the risk of osmotic diarrhea, and does so without contributing to gas production, which can reduce the likelihood of flatulence and abdominal pain (Figure 1).

#### 6.2.4. Lactose as a Prebiotic and Microbial Adaptation to Lactose Consumption

Addition of lactose to ex vivo cultures causes a rapid adaptation to lactose fermentation [95] including a shift towards taxa that produce β-galactosidase, a decrease in *Bacteroides* and an increase in lactic acid bacteria and *Bifidobacterium* [26]. Interestingly, though *Bacteroides* can produce β-galactosidase and ferment lactose, they do so less efficiently than *Bifidobacterium*, which likely drives this observed microbial shift (in part due to the acid production of *Bifidobacterium* that makes the pH less hospitable to *Bacteroides*) [26]. This shift towards lactose fermentation and bacteria that ferment lactose without gas production may be a mechanism for reducing symptoms associated with osmotic pressure and gas production. Several studies have investigated the hypothesis that low-level lactose consumption could alter the colonic microbiome to alleviate LI symptoms [74] (Table 3).

For example, a blinded study supplementing a low lactose diet with dextrose (on days 1–16 and 36–52) and lactose (on days 18–34, 0.3 g/kg of body weight per day, incrementally increasing by 0.2 g/kg every other day) to LI subjects found higher fecal β-galactosidase activity in the lactose supplementation period than during the dextrose phase (*p* < 0.001) [15]. This heightened β-galactosidase activity reduced quickly after lactose consumption ended. This β-galactosidase activity was likely due to the production of the enzyme by lactose-fermenting bacteria. Although post-lactose challenge breath hydrogen concentrations were reduced by approximately 50% during lactose consumption compared to dextrose periods, this change did not reach statistical significance. Within the same paper, a randomized study feeding either dextrose or lactose (0.6–1.0 g/kg of body weight per day for 10 days) followed by crossover to dextrose for 10 days in LI subjects (n = 20) indicated that subjects reported no severe symptoms and had a 50% reduction in flatulence after a lactose challenge during the lactose phase compared with the dextrose phase [15]. No significant changes in other symptoms, such as diarrhea and abdominal pain, were observed. Additionally, the breath hydrogen concentration measured after the lactose challenge was significantly lower following the lactose consumption period (9 ± 38 ppm/hour) than after the dextrose consumption period (385 ± 52 ppm/hour), indicating a significant digestive adaptation to lactose (*p* < 0.001) [15].

Another double-blind study feeding either 34 g of lactose or sucrose for two weeks to LI subjects (n = 46) found increased fecal β-galactosidase activity, decreased fecal pH and a reduction in breath hydrogen concentration after a lactose challenge in the lactose group compared with the sucrose group. Although symptoms associated with LI, excluding diarrhea, appeared to be less severe after lactose consumption, these differences were not statistically significant [14].

The investigations conducted by Hertzler et al. [15] and Briet et al. [14] both observed that continuous lactose intake was associated with an increase in fecal β-galactosidase activity and a decrease in breath hydrogen concentration after lactose challenge. This pattern is likely reflective of an increased population of lactose-metabolizing bacteria, such as *Lactobacilli* and *Bifidobacteria*, that efficiently ferment lactose without producing gas [11]. In a 12-week study assessing lactose adaptation in subjects with LNP (n = 25), participants consumed increasing doses of lactose, starting with 3 g twice daily and reaching up to 12 g twice daily. The intervention led to a significant rise in *Bifidobacterium* abundance and fecal β-galactosidase activity, while reducing post-lactose challenge breath hydrogen, indicating improved lactose digestion and tolerance [96].

Other studies also suggest colonic adaptations to lactose consumption. For example, a study feeding 33 g of lactose daily (from four servings of dairy food) for 21 days to girls aged 11–15 with LI (n = 14) found that breath hydrogen concentrations decreased significantly from the baseline to day 21 (*p* < 0.03) [97]. Only minimal LI symptoms were reported during the lactose challenges and the extended feeding period, but no significant changes in symptoms were observed from the start to the conclusion of the study [97].

Ito et al. also documented that a six-day intake of 15 g of lactose by LI males (n = 24) resulted in a reduction in the total count of *Bacteroides* and *Clostridium perfringens* and an increase in lactose-metabolizing bacteria, such as *Lactobacillus* compared to baseline. This study did not measure any LI symptoms or hydrogen breath concentrations [16].

In a comparative intervention involving LI participants (n = 23) and those with lactase persistence (n = 18), 25 g of lactose consumed twice daily for two weeks, followed by a 50 g lactose challenge, resulted in significant increases in *Bifidobacteria* levels for the LI group (0.72 log CFU per g of stool; *p* = 0.04) compared to the LP group. *Lactobacilli* counts increased numerically but not significantly in both groups. Additionally, there were numerical but non-significant reductions in hydrogen breath concentrations and lactose intolerance symptoms post-lactose challenge in the lactose maldigesters [98].

These studies collectively suggest that regular lactose consumption can induce a functional adaptation in the gut microbiome, potentially enhancing its lactose-metabolizing capabilities. These studies demonstrate colonic adaptation due to bacterial fermentation of lactose and its potential to act as a prebiotic. The consistent observation across these studies is the proliferation of lactose-metabolizing bacteria, such as *Bifidobacteria*, in response to lactose supplementation. Despite this bacterial growth, the resultant decrease in LI symptoms was often not statistically significant. These findings indicate a need for further investigation to understand the full extent to which continuous lactose supplementation could alleviate LI symptoms. Future research is also essential to solidify the proposed mechanism of symptom mitigation, hypothesized to be the enhanced growth of lactose-metabolizing bacteria, like *Bifidobacteria* and *Lactobacillus*, which can process lactose without the production of gaseous byproducts.

**Table 3:** Clinical studies on lactose supplementation to individuals with lactose intolerance.

Study	Intervention	Subject Count	Condensed Findings
[15]	Study 1: lactose (incrementally increasing from 0.3 to 1.0 g/kg of body weight over 17 days vs. dextrose control periods (crossover) Study 2: Lactose dose increased from (0.6 to 1.0 g/kg of body wt/day) vs. dextrose control in a 10-day crossover study	Study 1: 9; Study 2: 20	Study 1: Increased fecal β-galactosidase. Non-significant decrease in breath hydrogen post-lactose challenge. Study 2: No severe symptoms; significant reduction in flatulence and breath hydrogen during lactose phase in response to a lactose challenge.
[14]	17 g of lactose twice a day vs. sucrose control for 14 days	46 (lactose n = 24; sucrose n = 22)	Increased fecal β-galactosidase, decreased breath hydrogen after lactose challenge; non-significant decrease in symptoms.
[93]	Incremental lactose doses from 3 g to 12 g twice daily for 12 weeks	25	Bifidobacterium and fecal β-galactosidase activity increased; reduced post-lactose challenge breath hydrogen. Lactose well-tolerated.
[97]	33 g of lactose from 4 servings dairy food per day for 21 days	14	Significant decrease in breath hydrogen after lactose challenge; minimal LI symptoms without significant change over time.
[16]	15 g of lactose for six days	24	Decrease in Bacteroides and Clostridium; increase in Lactobacillus; no data on LI symptoms or hydrogen breath concentration.
[98]	25 g of lactose twice daily for two weeks	41 (23 LI, 18 LP)	Significant increase in Bifidobacteria for LI group; non-significant increase in Lactobacilli; non-significant reductions in symptoms and hydrogen breath after a lactose challenge.

## 7. Conclusions

This review has discussed LI, the genetic adaptations that are associated with LP, the main methods used to diagnose LI and prebiotic management of LI symptoms. Prebiotics may serve as a therapeutic to mitigate symptoms in people with LI. GOS can enhance the growth of colonic *Bifidobacterium*. Feeding GOS to subjects with LI enhances the relative abundance of *Bifidobacterium* and has been associated with symptom reduction. The mechanisms by which prebiotics can limit LI symptoms are not clear. However, the observed reduction may result from the fact that *Bifidobacterium* ferments lactose without producing gas, a major causative factor in typical LI symptoms. Furthermore, the consistent consumption of low levels of lactose may act as a prebiotic, promoting the growth of *Bifidobacterium* and other beneficial colonic bacteria. Additional studies investigating the specific mechanism of lactose utilization by lactose-metabolizing bacteria and the consequent mitigation of LI symptoms are needed to verify these observations.

## Figures and Tables

**Table 1:** Diagnostic approaches for LI with their benefits and limitations.

Diagnostic Approach	Type of Sample	Analyte or Focus of Detection	Expected Variation in Lactose Intolerant Individuals	Benefits	Limitations
Lactose Tolerance Test	Blood	Blood glucose levels at 30, 60, and 120 min post-lactose consumption	Glycemia remains steady	Minimally intrusive; economical	Results affected by subject-specific factors (e.g., gastric clearance)
Genomic analysis	DNA from blood	LCT gene mutations	Existence of known LNP-associated mutations	Can verify or rule out primary intolerance; minimally intrusive	Does not detect secondary intolerance
Lactase quantification in duodenal biopsies	Biopsy of post-bulbar duodenal mucosa	Enzyme lactase levels	Diminished or absent enzyme levels	Accurate diagnosis of lactase deficiency	Invasive; requires technical expertise during endoscopy; costly
Hydrogen Breath Test	Breath	Hydrogen levels before and after ingesting 25 g lactose challenge	Minimum 20 ppm rise in breath hydrogen from the baseline	Affordable; non-invasive; highly sensitive and precise; straightforward execution and interpretation	Time-consuming (3–6 h); requires avoiding a large array of foods and behaviors prior to testing to avoid false positives
Gaxilose Test	Blood and urine	Xylose concentration after gaxilose ingestion	Xylose levels not elevated indicating reduced lactase activity	Non-invasive; direct assessment of lactase activity; patient comfort	Relatively new test; lower specificity than lactose tolerance test

**Table 2:** Clinical studies on GOS supplementation of individuals with lactose intolerance.

Study	Treatment	Subject Count	Findings
[17]	Administered GOS or placebo (corn syrup) from 1.5 g/day to 15 g/day over 35 days	85 (GOS: 57; placebo: 28)	Trends toward symptom improvement such as less abdominal discomfort and bloating after a lactose challenge in the GOS group compared to placebo. Significantly lower flatulence after lactose challenge compared with placebo.
[12]	Administered GOS starting at 1.5 g/day, escalating to 15 g/day over 35 days without dairy vs. placebo control	85 (GOS: 57; placebo: 28)	GOS intake increased levels of lactose-metabolizing bacteria (Bifidobacteria, Faecalibacterium, Lactobacillus) relative to the baseline and to placebo. After dairy reintroduction for 1 month, microbial diversity was lower than baseline, likely due to the dominance of Bifidobacteria and Lactobacillus. A negative correlation was shown between increased Bifidobacterium and reduced cramping post-dairy.
[13]	Lower dose GOS (5 g twice daily for 10 days, 7.5 g twice daily for 20 days), higher dose GOS (7.5 g twice daily for 10 days, 10 g twice daily for 20 days) or placebo over 30 days	377 (lower dose: 126; higher dose: 121; placebo: 121)	Higher fecal Bifidobacterium species in both GOS groups. Decreased post-lactose challenge cramping and bloating in the pooled GOS groups. Tendency for decreased abdominal pain and gas movement in the GOS groups post-lactose challenge. Increased voluntary dairy consumption days 32 to 60 in GOS groups.
